# Catastrophic Early Failure and Fragmentation of a Modern Moderately Cross-linked Polyethylene Acetabular Liner

**DOI:** 10.1016/j.artd.2023.101161

**Published:** 2023-07-24

**Authors:** Nadim Barakat, James A. Browne, Quanjun Cui

**Affiliations:** Department of Orthopaedic Surgery, University of Virginia, Charlottesville, VA, USA

**Keywords:** Case report, Polyethylene wear, Early failure, Total hip arthroplasty, Exactech GXL

## Abstract

A 60-year-old man who underwent uncomplicated staged bilateral total hip arthroplasty for femoral head osteonecrosis presented with mechanical catching of his left total hip arthroplasty 3 years after index surgery. Radiographs revealed eccentricity of the left femoral head, concerning the failure of a modern moderately cross-linked polyethylene liner. Catastrophic polyethylene liner failure with significant wear, fragmentation, and femoral head abrasion was noted during revision surgery. The original liner and head were replaced, and the patient has exhibited no complications, pain, or difficulty ambulating 6 months postoperatively. This report highlights one potential novel mechanism for the failure of the Exactech Connexion GXL liner (Exactech Inc., Gainesville, FL), an implant recently reported to have a higher-than-expected failure rate, potentially due to insufficient packaging and increased oxidative processes.

## Introduction

Total hip arthroplasty (THA) is an incredibly effective treatment option for hip osteoarthritis and is considered one of the most cost-effective and successful surgeries in all of orthopaedics [1]. In fact, a recent meta-analysis of case series and reports of national joint replacement registries estimates 89% survival at 15 years and 58% survival at 25 years [2]. Etiologies for primary THA failure and the need for revision include but are not limited to dislocation, mechanical loosening, infection, and osteolysis [3,4]. Risk factors associated with revision of primary THA include younger age, male sex, and avascular necrosis [5]. Dislocation as a cause for primary THA failure is decreasing partially because of implant and technological improvements [4].

Cross-linked polyethylene acetabular liners are one example of implant technology that have dramatically improved the longevity and survivorship of THA. One meta-analysis of 8 studies confirmed that cross-linked polyethylene has substantially reduced radiological wear [6]. Meanwhile, a more recent randomized controlled trial with 10-year follow-up demonstrated that cross-linked polyethylene liners have significantly reduced wear and revision rates compared to conventional polyethylene [7]. Despite these potential benefits, there have been a few reports of early failures of cross-linked polyethylene acetabular liners, potentially due to differences in mechanical properties compared to conventional liners [[8], [9], [10], [11]].

Recently, there have been reports of early polyethylene failure and wear resulting in significant osteolysis with the recently recalled Exactech Connexion GXL liner (Exactech Inc., Gainesville, FL) [[9], [10], [11], [12]]. In order to contribute to the literature, we present a rare case of early unilateral failure of a staged bilateral THA using similar components but different acetabular polyethylene liners in a 60-year-old man due to catastrophic failure and fragmentation of the Exactech Connexion GXL liner, a modern moderately cross-linked polyethylene acetabular liner.

## Case history

The reporting of this study conforms to the Case Report guidelines [13]. The patient described in this report has provided written informed consent for the publication of details from his medical care.

**Figure 1:**
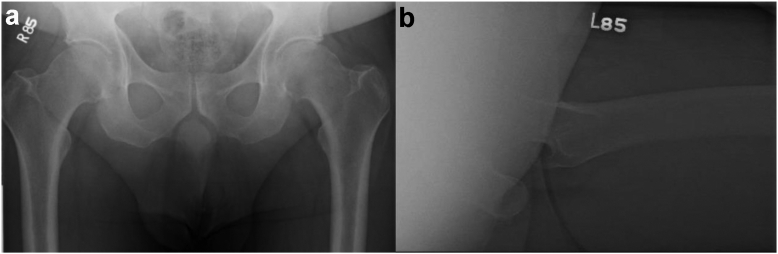
Preoperative left primary THA radiographs. (a) Anteroposterior (AP) pelvis and (b) left lateral radiographs demonstrate no acute fracture or bony malalignment. The left hip shows severe degenerative changes with joint space narrowing in addition to a small focus of superior femoral head avascular necrosis with collapse.

**Figure 2:**
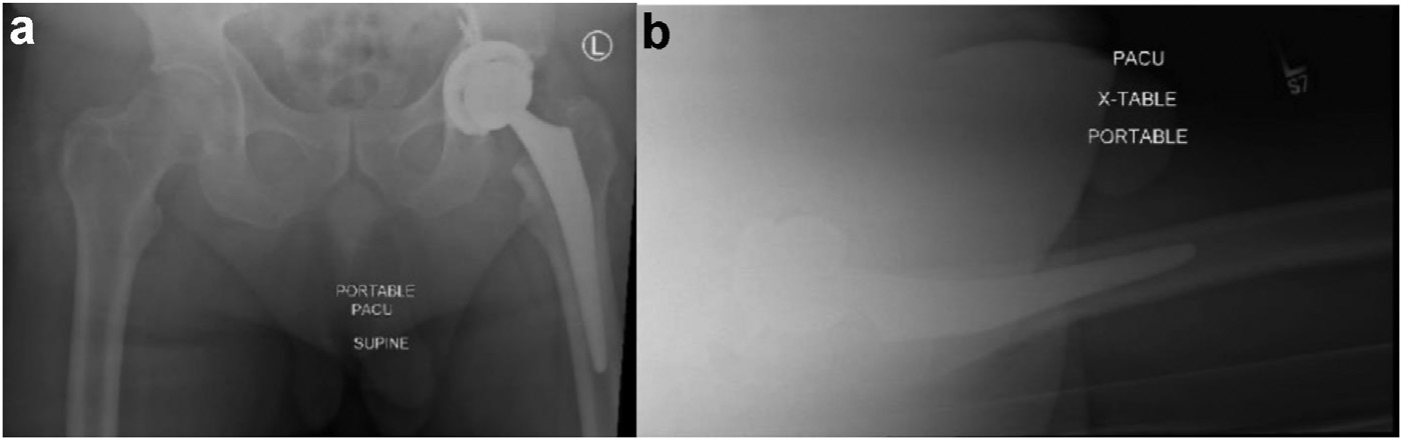
Left primary THA postoperative radiographs. (a) Anteroposterior (AP) pelvis and (b) left lateral radiographs demonstrate immediate postsurgical changes of left THA without periprosthetic fracture or malalignment. Sacroiliac joints are preserved with normal bone mineralization and no soft tissue abnormalities.

The patient is a 60-year-old Caucasian male with a body mass index of 38.4 kg/m^2^ and a medical history of hypertension, hyperlipidemia, and prediabetes with no smoking history. He presented in November 2018 for chronic worsening left hip pain that was no longer responsive to conservative measures. Radiographic imaging (Fig. 1) and an magnetic resonance imaging revealed severe degenerative changes consistent with osteonecrosis of the femoral head with osteoarthritis. In April 2019, the patient underwent left THA through the posterior approach. The implants used were a 4.6 mm thick, 58 × 40 mm with 5 mm lateralization Exactech Connexion GXL liner, Exactech Alteon tapered femoral stem with extended offset, 58 mm Exactech InteGrip acetabular shell, and a ceramic 40 mm Exactech Biolox Delta Novation femoral head, which is routinely used by the senior author when cup size reaches 54 mm. In consideration of the patient’s poor acetabular bone quality, which was appreciated intraoperatively, 3 Exactech Alteon screws were used to enforce the fixation. After obtaining postoperative radiographic imaging demonstrating reasonably positioned components (Fig. 2), the patient was discharged with no complications the day after the procedure.

At 2 and 8 months postoperative follow-up, the patient was ambulating well with no assistive device. On physical exam, the incision was well healed with no swelling or inflammation. The patient demonstrated mild pain with gentle range of motion (ROM) at 2 months postoperatively that improved to no pain at 8 months in addition to full strength on hip abduction, flexion, and extension. Radiographs at 2 and 8 months postoperatively continued to demonstrate well-fixed components without hardware complications.

**Figure 3:**
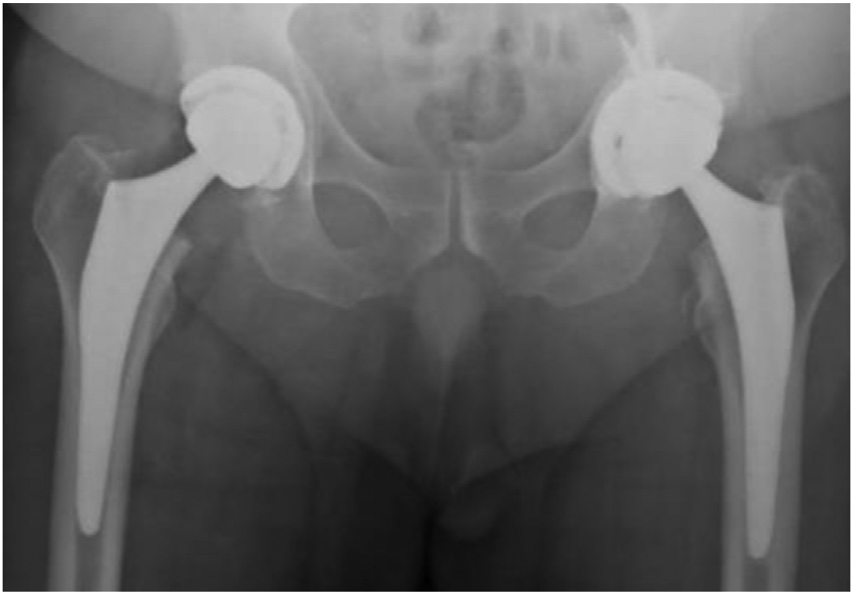
Bilateral THA postoperative radiograph. Anteroposterior (AP) pelvis radiograph demonstrates well-positioned bilateral THA without evidence of complications, fractures, or mechanical hardware failure immediately after right THA and 18 months after left THA.

In January 2020, the patient presented with chronically worsening right hip pain that was no longer responsive to conservative measures. Radiographic imaging and an magnetic resonance imaging revealed severe right hip osteoarthritis with edema and osteonecrosis of the right femoral head and neck and a well-positioned left THA 10 months postoperatively. In October 2020, the patient underwent right THA through the posterior approach for right hip osteoarthritis and osteonecrosis. The implants used were a 56 × 40 mm Exactech Alteon XLE vitamin E liner, Exactech Alteon tapered femoral stem, 56 mm Exactech Alteon acetabular shell, and a ceramic 40 mm Exactech Biolox Delta Novation femoral head. The Exactech Alteon XLE, a highly cross-linked vitamin E liner, had recently been acquired by the home institution and was used instead of the Connexion GXL liner to provide low wear while maintaining mechanical strength and reducing free radicals and oxidative degeneration. After obtaining postoperative radiographic imaging demonstrating well-positioned bilateral THAs without evidence of hardware failure (Fig. 3), the patient was discharged with no complications.

**Figure 4:**
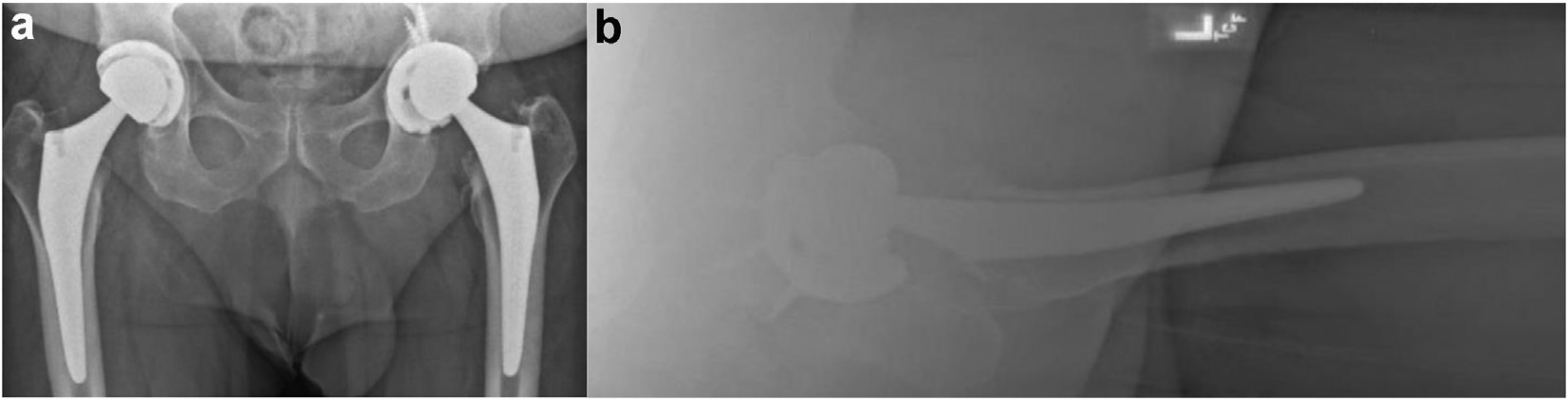
Preoperative left revision THA radiographs. (a) Anteroposterior (AP) pelvis and (b) left lateral radiographs of bilateral THA show the left femoral ball sitting asymmetrically within acetabulum, consistent with dislodgement of the polyethylene liner, 3 years postoperatively. (a) also shows well-fixed right THA without complication, 18 months postoperatively.

In April 2022 (3 years after primary left THA), the patient presented after acutely experiencing mechanical catching and grinding in the left hip in addition to mild left hip pain over the past few days. He had difficulty with ambulation but did not require an assistive device. The patient denied experiencing any inciting event, trauma, falls, excessive physical activity, instability, or dislocation, in addition to any recent redness, swelling, or inflammation near the incision site. On physical exam, there was no swelling or erythema near the incision; mild pain and mechanical catching were reproduced with full ROM and full strength of hip abduction, flexion, and extension. The patient showed no signs of systemic or surgical site infection on physical exam or in laboratory workup. Radiographs (Fig. 4) and a computed tomography scan showed a displaced left femoral head component with anterior and superior lateral migration, suspicious for polyethylene liner failure.

**Figure 5:**
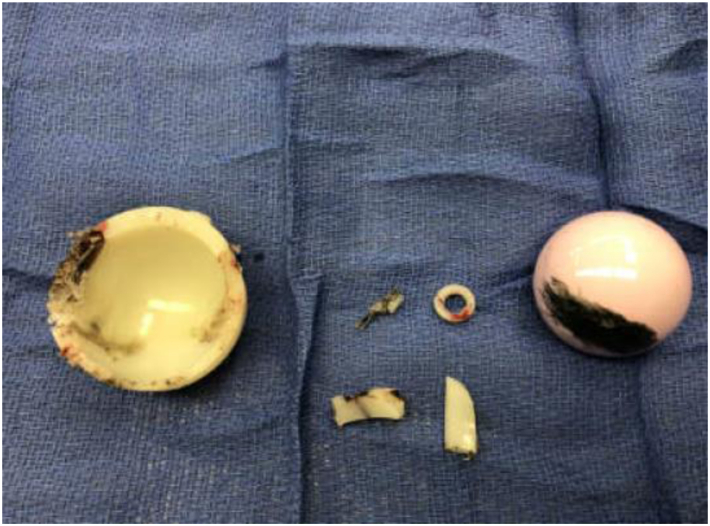
Explanted components. Retrieved polyethylene liner and femoral head demonstrate catastrophic failure of the polyethylene liner with significant wear and fragmentation in addition to abrasion of the femoral head.

The patient underwent revision THA through the posterior approach for acute hardware failure due to polyethylene liner wear with mechanical problems of the left THA, 3 years after implantation. Upon exposure, there were fragmented polyethylene liner pieces in the joint. In addition, there was minimal head and cup articulation at the fractured site of the liner, resulting in metallosis and dark-colored fluids. Also, there was a linear scratch on the surface of the femoral head and significant wear and failure of the polyethylene liner (Fig. 5). The original 40 mm with 5 mm lateralized polyethylene liner and 40 mm ceramic femoral head were explanted and replaced with a 40 mm nonlateralized Exactech Novation XLE neutral liner and a ceramic 40 mm Exactech Biolox femoral head with satisfactory intraoperative stability. The Exactech Novation XLE neutral liner is a highly cross-linked vitamin E-enhanced polyethylene liner and was used to replace the fragmented Connexion GXL liner because of its ability to reduce free radicals and oxidative degeneration. After obtaining postoperative imaging showing well-positioned bilateral THAs, the patient was discharged with no complications.

**Figure 6:**
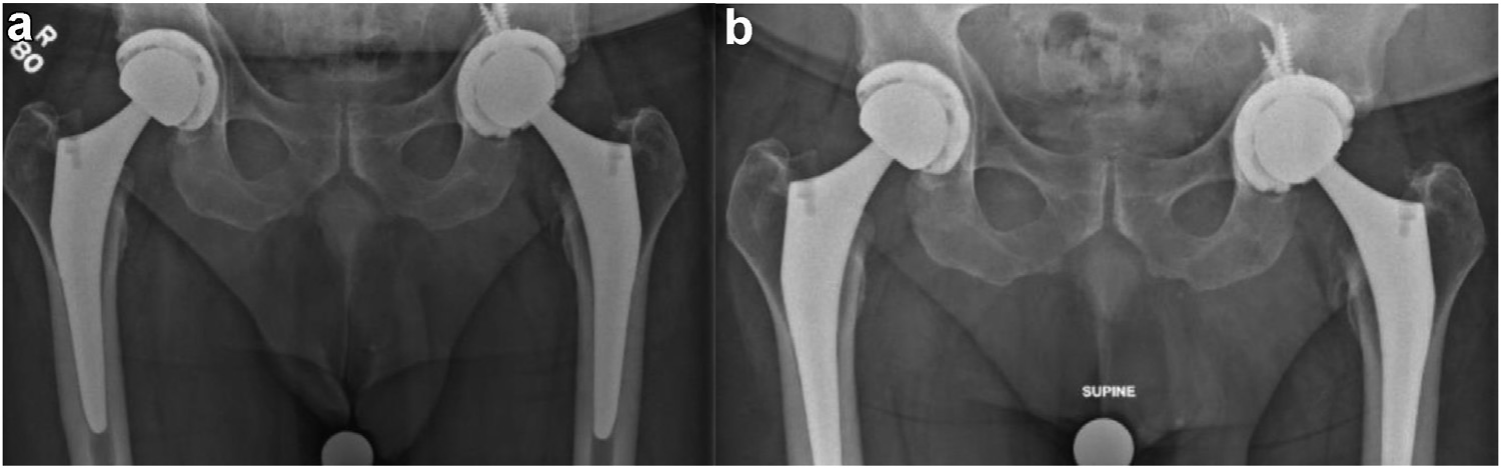
Postoperative left revision THA radiographs. Anteroposterior (AP) pelvis radiographs (a) 1 month and (b) 6 months postoperatively of left revision THA show well-aligned bilateral THA with no signs of complication.

In May and October 2022, the patient presented for his 1- and 6-month follow-up for left revision THA. Radiographs revealed well-aligned bilateral THA with no signs of hardware failure (Fig. 6). He was not experiencing pain or instability and was able to ambulate without any assistive devices. The incision site had no signs of inflammation, and the patient demonstrated full ROM and strength on hip abduction, adduction, flexion, and extension.

## Discussion

The patient in this report underwent early revision THA due to early catastrophic failure and fragmentation of an Exactech Connexion GXL moderately cross-linked polyethylene liner. While this patient did exhibit factors that could be associated with revision of primary THA such as male sex, osteonecrosis, and obesity [14,15], we do not feel that these potential risk factors explain the findings of the explanted polyethylene liner in Figure 5. Although a large femoral head in a small acetabular cup was used to reduce the risk of dislocation and could potentially lead to excessive wear of the cross-linked polyethylene liner, this factor is also an unlikely explanation, as prior research has suggested wear of cross-linked polyethylene liners is independent of femoral head size [16]. While we are unable to determine the cause of the polyethylene liner failure from this singular case, to our knowledge, there are 3 other publications that have reported complications with this specific polyethylene liner [[9], [10], [11]]. However, these reports all document increased wear and osteolysis, whereas our report appears to be the first to report catastrophic liner breakage. To our knowledge, no publication has shown such catastrophic failure and fragmentation of the polyethylene liner as seen in the patient presented in this report.

Thomas et al. reviewed their institutional database from 2009 to 2019 for patients presenting with osteolysis in the setting of a THA utilizing the Exactech Connexion GXL polyethylene liner. They found 9 of the 12 patients in their investigation underwent revision surgery for early polyethylene wear and secondary osteolysis at an average postoperative time of 55.9 months (range: 12-120 months). In addition, they reviewed the Manufacturer and User Facility Device Experience database from 2009 to 2019 and found 22 reported cases of wear-related failure. They suggested the locking mechanism or manufacturing characteristics as the potential cause for failure [9]. Kahlenberg et al. published a case series in which they identified 5 cases of severe polyethylene wear and osteolysis that occurred within 5 years of primary THA using the Exactech Connexion GXL liner in 204 patients. They postulated that a variation in the processing of some of the liners was responsible for these failures [10]. Yakkanti et al. compared a study group that underwent THA with the Exactech Connexion GXL liner with another group that used another moderately cross-linked polyethylene liner. The study group had a significantly higher wear rate above the osteolysis threshold, which was verified radiographically and macroscopically. They also proposed that the polyethylene itself or its manufacturing played a role in the high wear rates they had observed [11].

There have been reports of reasonable outcomes using this liner. In a letter to the editor, Godoy-Monzon and Cid-Casteulani reported that the Exactech Connexion GXL liner had demonstrated success in 2 of their midterm studies [17]. In a prospective study of 104 patients, there were no revisions 5 years after the index surgery and only 7 revisions 9 and a half years after the index surgery. Their studies yielded a 93% survivorship considering all causes for revision [18,19].

Exactech Inc. recently issued a recall for several implants including the Connexion GXL liner for insufficient packaging. They have disclosed that many inserts were packaged into vacuum bags lacking a secondary oxygen barrier layer, which normally augments oxygen resistance and prevents oxidation [20]. Consequently, this ineffective packaging process may have resulted in increased oxygen diffusion and oxidation of the inserts over time. These oxidative processes and degradation may explain the accelerated wear and failures seen in these mispackaged polyethylene liners [21].

## Summary

This report of a catastrophic early failure and fragmentation of a modern moderately cross-linked polyethylene liner appears to be a novel mechanism of failure of the Exactech Connexion GXL liner. This failure may be related to the recent Exactech recall, which indicated certain liners were insufficiently packaged, predisposing them to high levels of oxygen exposure and consequent oxidation. Surgeons should be aware of the concerns with this implant as well as potential failure mechanisms.

## Conflicts of interest

J.A. Browne serves as a board or committee member of the American Association of Hip and Knee Surgeons, American Joint Replacement Registry (American Academy of Orthopaedic Surgeons), Southern Orthopedic Association, Hip Society, and Knee Society; received royalties and serves as a paid consultant to Enovis; serves on editorial or governing board and received financial support from the Journal of Arthroplasty; serves as a paid consultant to OsteoRemedies and Kinamed; has stock or stock options held in RadLink; and received financial support from Elsevier and the Journal of Bone and Joint Surgery. Q. Cui received research support from Depuy and Exactech; received financial support from Elsevier; serves on editorial or governing boards of the Journal of Arthroplasty and the Journal of Orthopedic Research; and serves as a board or committee member for the Association Research Circulation Osseous. None of the following authors or any immediate family members has received anything of value from or has stock or stock options held in a commercial company or institution related directly or indirectly to the subject of this article: N. Barakat.

For full disclosure statements refer to https://doi.org/10.1016/j.artd.2023.101161.

## Informed patient consent

The author(s) confirm that written informed consent has been obtained from the involved patient(s) or if appropriate from the parent, guardian, power of attorney of the involved patient(s); and, they have given approval for this information to be published in this case report (series).

## Supplementary data

Conflict of Interest Statement for Cui

Conflict of Interest Statement for Browne

Conflict of Interest Statement for Barakat
